# Pilot Study of the Delivery of Microcollimated Pars Plana External Beam Radiation in Porcine Eyes: 270-Day Analysis

**DOI:** 10.1155/2012/615214

**Published:** 2012-07-11

**Authors:** Rishi P. Singh, E. Mark Shusterman, Darius Moshfeghi, Ronald Danis, Michael Gertner

**Affiliations:** ^1^Cole Eye Institute, Cleveland Clinic Foundation, Cleveland, OH 44195, USA; ^2^Oraya Therapeutics, Newark, CA 94560, USA; ^3^Stanford University, Palo Alto, CA 94304, USA; ^4^University of Wisconsin, Madison, WI 53706, USA

## Abstract

*Objective*. To determine the dose response and toxicity threshold of micro-collimated X-rays delivered to porcine maculae by a stereotactic radiosurgical system after 270 days. *Methods*. Twelve eyes of six Yucatan mini-swine were randomized to receive up to 90 Gy to the retina, using an office-based trans-pars plana delivery system. To determine the safety profile of this radiation delivery, ophthalmic examination, fundus photography, fluorescein angiography (FA), and spectral domain optical coherence tomography (SD-OCT) were obtained at multiple time points up to 270 days post treatment. *Results*. No abnormalities were noted on external examination. Cataracts were noted in 4 of 12 eyes. Dose and time-dependent changes were noted on fundus examination, FA, ICG and SD-OCT. No significant abnormalities were seen in the control, 16 Gy or 24 Gy groups using any modality. Histopathology revealed a dose response effect with no discernable lesions in the 16 Gy group. *Conclusion*. The X-ray delivery system precisely targets the porcine retina in vivo with little effect on surrounding structures. No ophthalmic or intracranial adverse effects were noted at clinically relevant doses at 270 days following radiation delivery.

## 1. Introduction

Age-related macular degeneration (AMD) is the leading cause of blindness in the Western world for those 50 years of age and older [1]. Neovascular, or exudative, AMD is associated with choroidal neovascularization (CNV) and thus responsible for more rapid and severe vision loss than its nonexudative counterpart. The introduction of anti-VEGF agents has revolutionized the treatment of this condition, dramatically improving visual outcomes with monthly intravitreal injections [2, 3]. While the blockade of VEGF is critical in the prevention of angiogenesis and also serves to decrease the vascular permeability of existing CNV, it does not lead to regression of the mature vessels found in CNV or prevent the onset of inflammation and fibrosis that lead to scar formation. As a result, neovascular AMD has shifted from a rapidly progressive disease to a chronic ailment: repeated intravitreal injections of anti-VEGF are needed to preserve vision, with no guarantee of avoiding scar formation.

Ionizing radiation has been proposed as a treatment of neovascular AMD, as it inhibits new blood vessel growth [4] and has a particular impact on vascular endothelial cells [5]. One possible benefit of radiation treatment of AMD in conjunction with anti-VEGF injections is the synergistic inhibition of neovascularization [6]. Unlike anti-VEGF therapies, radiation also has antifibrotic properties, making it an even more attractive adjunct. Another theoretical benefit of combination therapy is the potential for less frequent anti-VEGF retreatment, reducing the social and financial burden, as well as the risk of endophthalmitis, retinal tears, retinal detachments, vitreous hemorrhage, and hypotony that can result from repeated injections [7, 8].

We recently published our interim data evaluating the same radiotherapy system [9]. This analysis found that no abnormalities of external structures were noted at Day 90. Fundus evaluation revealed no abnormalities at the 16 or 24 Gy doses. Beginning at day 30, circular pale retinal lesions with sharp margins were noted in the maculae of the eyes that received 42, 60, and 90 Gy. These areas had corresponding regions of choroidal nonperfusion and retinal capillary dropout. The study concluded that transscleral stereotactic radiation dosing of porcine eyes demonstrated no apparent clinical abnormalities in doses at or less than 24 Gy at least through day 90. A longer follow-up period was needed, especially for the lower dose animals, to determine adverse event rates, since the effects of radiation are not apparent for some time after treatment. Finally, although spectral domain optical coherence tomography (SDOCT) was performed to show “in vivo” morphometric changes, histological analysis could be performed in the last stages of the study to truly define the treatment beam path and to determine whether the SDOCT findings correlated with histology.

The purpose of this study was to evaluate the effect of transconjunctivally administered external beam radiation on the retina of Yucatan miniswine through day 270. Ophthalmic examination, fundus photography, fluorescein angiography (FA), indocyanine green (ICG) angiography, and SDOCT images were obtained to document the potential adverse events of escalating doses of radiation over time. Computed tomography (CT), magnetic resonance imaging (MRI), and histology were performed at the completion of the study period.

## 2. Study Design and Treatment

After the Institutional Animal Care and Use Committee (IACUC) approval was obtained, Yucatan mini-swine, procured from S&S Farms (Ramona, CA), were used for this study, as their eyes resemble human eyes in structure, size, and vascular pattern [10]. The study adhered to the ARVO statement on the treatment of animals. The animals were determined to be healthy at arrival and were quarantined for eight days before being enrolled in the study. 

**Figure 1:**
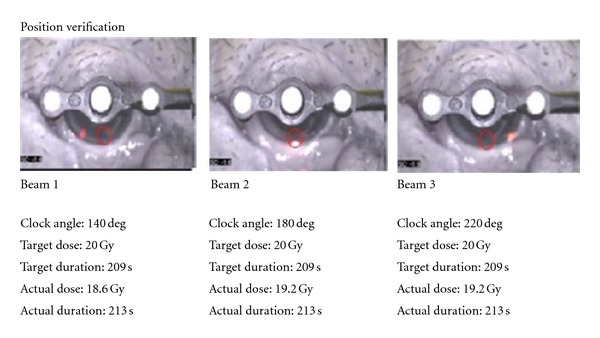
The figure demonstrates the beam positions on the sclera of the animal's right eye. Each beam passes through the area of the porcine eye akin to the human pars plana. A 60 Gy dose was delivered in this example.

Twelve eyes of six animals were randomized to six different dose groups: control, 16 Gy, 24 Gy, 42 Gy, 60 Gy, and 90 Gy. Radiotherapy was delivered in a single session using a low-energy stereotactic device (IRay: Oraya Therapeutics, Inc., Newark, CA) that aims multiple beams at a single retinal target, as previously described in an interim report [9]. Figure 1 demonstrates the X-ray beam entry points on the sclera.

Ophthalmic examinations (slit lamp and indirect ophthalmoscopy), including intraocular pressure measurements (Tono-Pen XL, Medtronic ENT, Jacksonville, FL), fundus photography, fluorescein angiography (FA), and spectral domain optical coherence tomography (Cirrus HD-OCT, Carl Zeiss Meditec, Dublin, CA) were performed on each eye prior to treatment and at days 7, 30, 90, 150, 210, and 270. Ophthalmic observations of both eyes were scored and recorded according to the McDonald-Shadduck system [11]. All imaging was examined by a masked grader (RPD) and evaluated for abnormalities. Indocyanine green angiography (ICG) was done at days 90 and 270. Computed tomography (CT) and magnetic resonance imaging (MRI) were performed at day 270. The animals were euthanized thereafter, and the orbital contents were then evaluated for histopathologic changes.

## 3. Results

### 3.1. External and Anterior Segment Findings

All swine gained weight and there was no mortality during the study. Ophthalmic examinations revealed no significant external findings during the course of the study. Intraocular pressure (IOP) varied throughout the study in all eyes. No significant trends in IOP were noted with respect to treatment or time.

**Figure 2:**
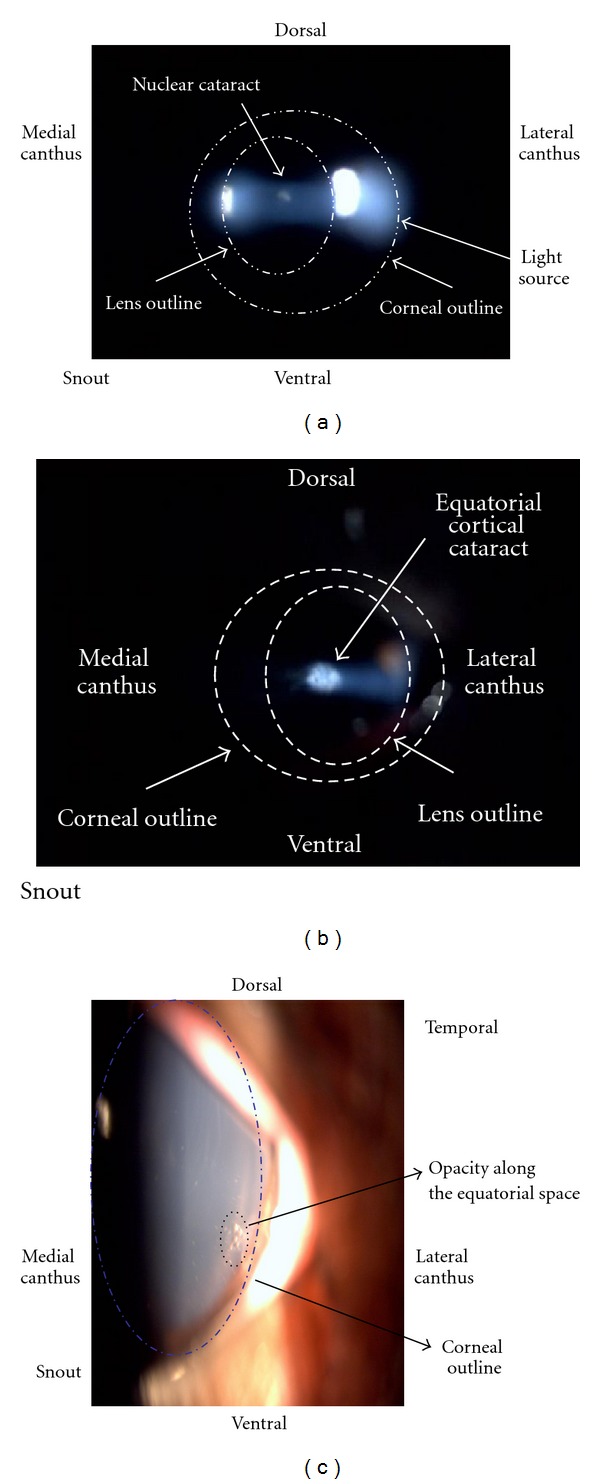
(a) Nuclear cataract found in the 0 Gy animal at day 205. At day 270, no significant worsening was noted. (b) and (c) Cortical lens changes seen in the 90 Gy animal at day 270 by slit lamp examination and by external ocular photography.

Three of the twelve eyes developed nuclear cataracts at day 210 or later. These eyes spanned the gamut of treatment groups, receiving 90 Gy, 16 Gy, and no treatment. One additional eye developed a focal, cortical cataract with 60 Gy treatment. Photos of the cataracts are shown in Figures 2(a)–2(c). While cataracts were detected at the 60 and 90 Gy radiation doses, as well as a 16 Gy eye, a cataract was also present in a control eye, suggesting that the cataracts in the zero and 16 Gy groups may have been age-related. The cataracts noted at the 60 and 90 Gy doses appeared to be relatively more proximate to the X-ray beam paths, located along the nasoventral aspect of the eye, while the cataracts seen at the control and 16 Gy doses seemed to be more central, further suggesting an etiology other than radiation. 

### 3.2. Fundus, Fluorescein, and Indocyanine Green Findings

**Figure 3:**
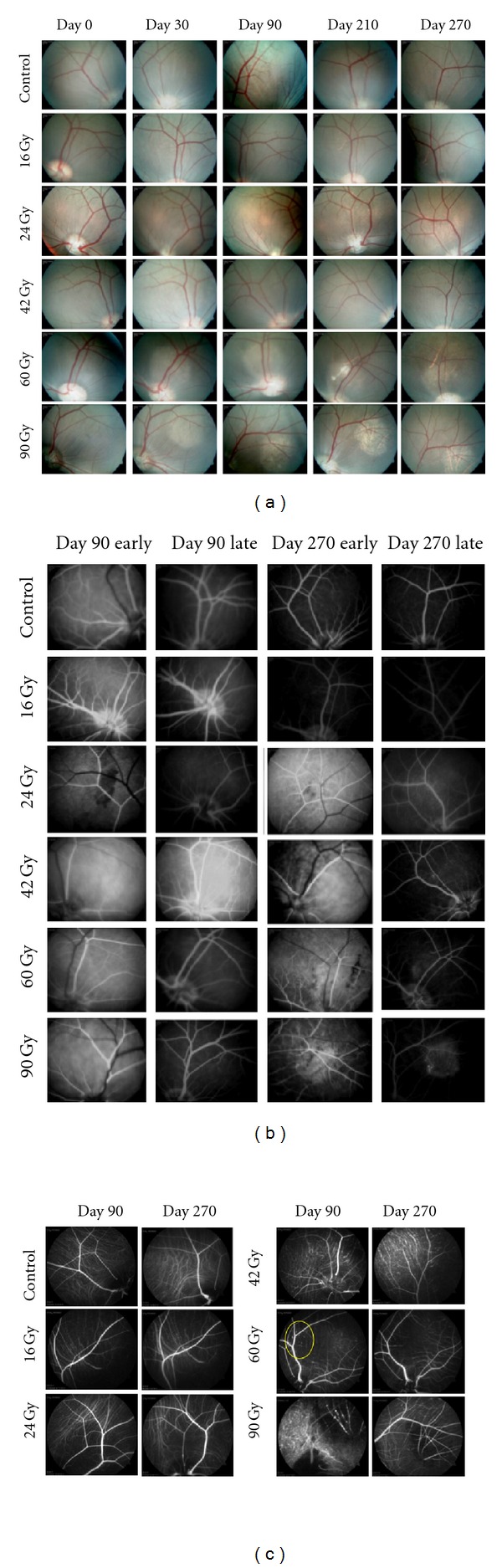
(a) Montage images of fundus photographs showing dose-dependent changes over the time course of the study. In the doses above 24 Gy, cotton wool spots, dot hemorrhage, and eventual atrophy were seen with depigmentation. (b) Montage images of fluorescein angiograms showing dose-dependent changes over the time course of the study. In the doses above 24 Gy, there was early retinal capillary changes with eventual pruning of vessels and late staining consistent with damage to the retinal pigment epithelium and retina vasculature. (c) ICG images demonstrating choroidal capillary obliteration at the 60 Gy and 90 Gy doses.

Fundus photography and FA showed a dose-dependent and time-dependent evolution of treatment spots (Figures 3(a) and 3(b)). At day 30, retinal whitening was noted in both 90 Gy eyes and one of two 60 Gy eyes. At day 90, the 90 Gy group developed cotton wool infarcts, retinal capillary drop out, and choroidal ischemia. Both 60 Gy eyes and one 42 Gy eye developed pigmentary changes without definite FA changes. Pigmentary changes were severe in the 60 Gy eye with previous retinal whitening and mild in the others. By day 210, the second 42 Gy eye also demonstrated RPE changes, as well as delayed choroidal filling. Both 60 Gy eyes had FA changes—obvious retinal capillary closure and delayed choroidal perfusion in one, late staining and leakage in the other. At day 270, the less-affected 60 Gy eye developed retinal vascular caliber irregularities, while both 42 Gy eyes continued to show mild pigmentary changes, one with retinal capillary and choriocapillaris loss. No changes were found in the 16 Gy and 24 Gy groups in the first five months. At day 210, one 24 Gy eye showed sheathing and irregularity of retinal arterioles, followed by similar changes in one 16 Gy eye at day 270. No abnormalities were noted on any images for the control group.

Indocyanine green Angiography showed choroidal ischemia beginning at day 90 for the 90 Gy group and day 270 for the 60 Gy group (Figure 3(c)). No ICG abnormalities were noted for the other groups. In all cases, regions outside the treatment area appeared normal on FA and ICG.

### 3.3. Optical Coherence Tomography Findings

**Figure 4:**
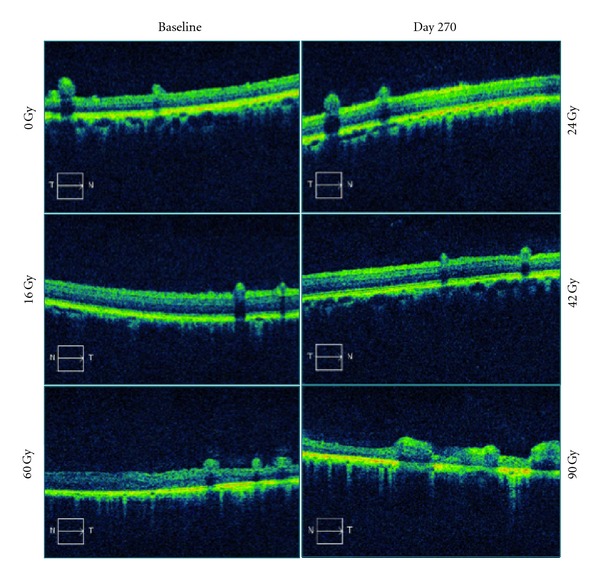
Representative OCT montage of a 24 Gy and a 90 Gy animal, demonstrating dose-dependent treatment effect at day 270. In the 24 Gy swine, no obvious defects were seen with regard to retinal thinning or RPE hyperreflectivity. In 90 Gy animal, there is a dose-dependent decrease in retinal thickness and an increase in the RPE hyperreflectivity indicating significant retinal scarring. No findings were noted at 0 or 16 Gy, and progressively worsening retinal injury was seen at the 42, 60, and 90 Gy doses.

Examination by spectral domain OCT also showed dose- and time-dependent changes (Figure 4). Retinal thinning was observed in the 90 Gy group as early as day 7 in one eye, and day 30 in the other, progressing to severe atrophy over subsequent examinations. The 60 Gy group showed questionable thinning in the same timeframe, but also developed severe atrophy by day 210. In the 42 Gy eyes, mild retinal thinning was first noted at days 150 and 210. No OCT changes were found in the 24 Gy, 16 Gy, or control eyes.

Computed tomography (CT) and magnetic resonance imaging (MRI) findings. On CT, performed at day 270, there was no evidence of bony abnormalities suggestive of radiation osteonecrosis. The calvarium, skull base, and orbits appeared normal and did not have any sclerotic or lucent changes. No intraorbital, soft tissue, or bony mass was present in 4 of 6 animals. An ill-defined soft tissue lesion with internal calcifications was seen in the posterior musculature and subcutaneous fat of the right neck of one animal, consistent with multiple injection granulomas. Another mini-swine had changes suggestive of mild, bilateral degenerative joint disease in the temporomandibular joints.

On more detailed soft tissue analysis by MRI, neither optic neuritis nor radiation necrosis within brain parenchyma was found for any animal. All animals had T1-bright signal intensity in the calvarium, immediately adjacent to the inferior margin of the frontal sinuses on the postgadolinium sequences. Three of six mini-swine had a similar signal intensity bilaterally in the skull base, adjacent to the inferior margin of well-developed sphenoid sinuses. These findings were deemed artifacts, as they are most consistent with failure of fat suppression at the bone-air interface. Visualized bone marrow was otherwise normal in signal intensity in all animals. 

### 3.4. Histopathological Findings

**Figure 5:**
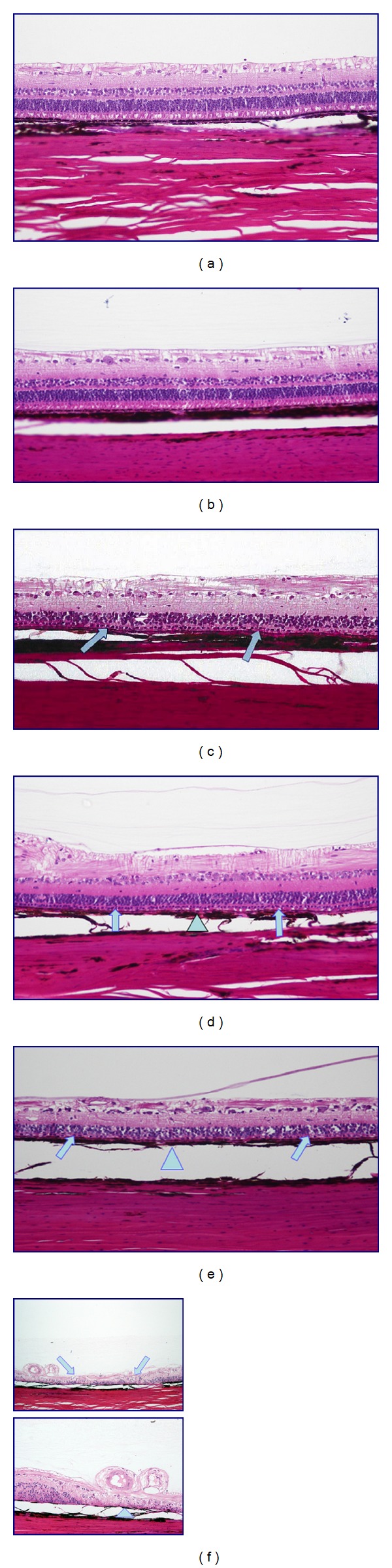
Retinal, RPE, and choroidal histological changes in all dosage groups through the target lesion site. (a) Control, (b) 16 Gy, and (c) 24 Gy demonstrate no discernible retinal, RPE, or choroidal changes. (d) 42 Gy shows a broad based lesion with photoreceptor, outer nuclear layer atrophy (arrow), and mild to moderate RPE damage (triangle). (e) 60 Gy shows a more impressive lesion with loss of photoreceptors and outer nuclear layer attenuation (arrow) and moderate RPE attenuation (triangle). (f) 90 Gy demonstrates distinct areas of damage involving the entire thickness of the retina (arrow) with RPE and choroidal disruption (triangle).

Histopathological evaluation of the eyes yielded a range of damage that correlated with the amount of radiation (Figures 5(a)–5(f)). Both eyes in the 90 Gy group showed a distinct lesion with loss of the photoreceptors and outer nuclear layer (ONL) throughout, with involvement of the inner nuclear (INL) and ganglion cell layers (GCL) centrally. There was extensive disruption and atrophy of the RPE, with only focal areas of choroidal pigment loss. Deeper choroidal and scleral tissue was unaffected, as well as retinal vessels within the lesion. Eyes in the 42 Gy and 60 Gy groups also showed diffuse photoreceptor loss and ONL changes within distinct lesions. However, the INL and GCL were unaffected and only mild to moderate RPE attenuation was found. Eyes that had received 16 Gy, 24 Gy, or no treatment showed no discernible lesions.

## 4. Discussion

This randomized, dose-escalation study was designed to evaluate delivery of low voltage external beam radiation to the eye of Yucatan mini-swine, in doses escalating from 0 to 90 Gy in three convergent beams. This trial demonstrated dose-dependent delivery of radiation in mini-swine in a precise fashion with little effect on surrounding structures. The extremely high dose of 90 Gy served to establish the system's performance in terms of accurate beam convergence. Doses above 24 Gy demonstrated clear retinal and choroidal changes as measured by FA, ICG, and SDOCT with little collateral damage to structures through which the X-ray beams passed. In addition to the clinical findings, postmortem histology confirmed that eyes receiving more than 24 Gy of radiation developed loss of photoreceptors and ONL within a well-defined lesion, with additional layers, and the RPE being affected as the dose escalated. No histologic changes were noted in the 16 Gy, 24 Gy, or the control groups. The initial 90-day follow-up period only demonstrated radiation effects in the 60 and 90 Gy groups. Because of the slow progressive nature of radiation damage, it was critical to perform this 270-day follow-up to monitor for after effects across all doses.

No significant external or corneal abnormalities were noted and the IOP was unaffected. Importantly, there was no discernible effect on the sclera of any eye at any dose, even at very high scleral exposures, as noted in Table 1. While cataracts did develop in 4 of 12 eyes, only one was focal, and cortical in nature, and could be attributed to the 60 Gy of radiation administered, well above clinically relevant levels. The treatment was also well tolerated regionally, with no evidence of radiation necrosis or optic neuritis on either head CT or MRI of all animals at day 270.

Retinal lesions were visualized on fundus photography, and appeared first in the 90 Gy and 60 Gy groups, and then in the 42 Gy group; these lesions may represent loss of the photoreceptor layer, as noted in work by Amoaku et al. [12] OCT of these regions showed well-demarcated transition zones from a thicker, normal photoreceptor layer to an area of receptor thinning and loss. The lesions are extremely well circumscribed, of approximately the same size as the beam spot delivered by the IRay device onto the retina, and located at the targeted zone, superior and temporal to the optic nerve. These observations provide a strong signal of localized radio effect on the central, avascular area of the affected eyes, corresponding to the human macula. Equally importantly, the retinal lesions show no indication of enlargement over time, providing anatomic evidence of a highly localized, precise delivery of radiation using the IRay system. Post-mortem histopathological examination confirmed the dose-response findings observed on imaging throughout the study. The periodic comprehensive examinations performed in live animals during the course of this study provided valuable information pertaining to ongoing anatomic changes in the eye produced by radiation exposure. These observations were consistent with findings seen in the CLA001 study previously conducted by Oraya, which served as the precursor to this investigation [13]. In that study, photoreceptor loss was demonstrated histologically at the target region of a porcine eye irradiated with 60 Gy. Of note, a similar hypopigmented lesion was seen in that eye during fundus photography at a month and more following radiation.

An added advantage of the porcine model used for this study is the similarity of the anterior chamber anatomy of the mini-swine to that of the human. The relatively large lens size of the mini-swine allowed monitoring of the dose-escalation response on the lens. Two high-dose eyes were seen to have small focal cataracts at the later follow-up time points. However, the presence of what appeared to be a focal cataract in a control eye at the day-210 assessments suggests the possibility of radiation-unrelated natural aging effects in the higher dose eyes. Furthermore, it must be emphasized that there are significant differences between the porcine and the human eye in terms of the very large size of the porcine lens relative to the overall diameter of the globe. This larger size does not accommodate the same pars plana beam path as is utilized in treatment in the human eye. Considerably more radiation would be delivered to the porcine lens due to this anatomical difference than would be seen in human clinical use.

In summary, stereotactic radiation delivery to the macula of mini-swine was accomplished with accurate targeting using Oraya's IRay device. Dose- and time-dependent retinal changes were noted. At doses that have been previously employed in clinical trials, no adverse events of significance were seen in the eye, orbit, or brain. These results, coupled with Oraya's CLA001 study [13], the Monte Carlo treatment simulation of the IRay system [14], and the study of Ávila et al., describing delivery of radiation to the macula using an epiretinal brachytherapy approach [15], suggest that Oraya's IRay device is able to place X-rays onto the macular target in a precise and localized manner, without apparent adverse effects at the clinically proposed doses of 16 and 24 Gy.

## Figures and Tables

**Table 1:** IRay Treatment Dosing Schema of Yucatan Mini-Swine.

Animal No.	Total Dose OD (Gy)	Axial Length OD (mm)	Single Beam Retinal Dose OD (Gy)	Single Beam Scleral Dose OD (Gy)	Total Dose OS (Gy)	Axial Length OS (mm)	Single Beam Retinal Dose (Gy)	Single Beam Scleral Dose (Gy)
1	60	17.39	20.00	43.8	24	17.54	8.00	17.7
2	90	17.55	30.00	66.2	42	17.40	14.00	30.7
3	24	17.16	8.00	17.3	0	17.91	0.00	0.0
4	16	17.00	5.33	11.5	60	17.08	20.00	43.2
5	0	17.70	0.00	0.0	90	17.72	30.00	66.7
6	42	17.93	14.00	31.5	16	17.71	5.33	11.9
